# Bridging migraine and psychosis: a neuropsychiatric review of shared dopaminergic mechanisms

**DOI:** 10.3389/fneur.2025.1577146

**Published:** 2025-05-09

**Authors:** Rithin Jacob Antony Rajan, Roshini Esther A

**Affiliations:** ^1^Faculty of Medicine, Alte University, Tbilisi, Georgia; ^2^Department of Biopharmaceutical Technology, University College of Engineering, BIT Campus, Chennai, Tamil Nadu, India

**Keywords:** neurology, psychiatry migraine disorder, psychotic disorders, dopamine receptors, neuroimaging, antipsychotic agents, migraine with aura, dopamine antagonists

## Abstract

Migraine is increasingly recognized as a disorder with both neurological and psychiatric dimensions, with increasing evidence suggesting a potential link to psychotic symptoms through shared dopaminergic dysfunction. Both conditions exhibit dopamine receptor hypersensitivity, neurotransmitter imbalances, and altered cortical excitability, raising the question of whether migraineurs, particularly those with aura, may be more susceptible to transient psychotic-like symptoms. This review explores the neurobiological overlap between migraine and psychosis, focusing on shared pathophysiological mechanisms, neuroimaging findings, and clinical evidence. Neuroimaging studies have revealed that migraineurs exhibit altered dopamine receptor binding and reduced synaptic dopamine during attacks, whereas psychotic disorders are characterized by sustained dopaminergic hyperactivity. These observations support the hypothesis that dopaminergic dysregulation may predispose migraineurs to sensory–perceptual disturbances that occasionally resemble psychotic symptoms. Importantly, this overlap does not imply that migraine leads to psychosis but rather suggests that shared neurochemical dysfunction may transiently modulate perception. Future research should clarify this interaction to improve the early identification and management of neuropsychiatric manifestations in migraine patients.

## Introduction

Migraine is a complex neurological disorder characterized by recurrent, severe headache attacks, often accompanied by sensory disturbances such as nausea, vomiting, photophobia, and phonophobia (1, 2). In addition to these classical features, migraine has also been associated with occasional neuropsychiatric manifestations, including transient psychotic-like symptoms such as perceptual distortions, hallucinations, and paranoia, especially in individuals experiencing migraine with aura (3, 4). In addition to its role as a primary headache disorder, migraine is increasingly recognized for its complex neuropsychiatric associations, particularly involving dopaminergic dysfunction (2, 5, 6). Dopamine plays a critical role in migraine pathophysiology, influencing sensory processing, pain modulation, and autonomic function (7, 8). Clinical and pharmacological evidence suggests that migraineurs exhibit hypersensitivity to dopamine agonists, which can provoke yawning, nausea, and vomiting (9, 10). Conversely, dopamine antagonists have been shown to alleviate migraine symptoms, further supporting the role of dopamine dysregulation in migraine (11). Genetic studies have also linked variations in the D2 dopamine receptor to increased susceptibility to migraine (7, 9, 12, 13). Importantly, while the clinical manifestations of migraine are primarily sensory and autonomic, emerging evidence suggests that dopaminergic dysregulation may transiently affect higher-order cognitive and perceptual processing, contributing to psychotic-like symptoms in some cases (3, 4).

Dopaminergic dysfunction is also a well-established feature of psychotic disorders, including schizophrenia, where excessive dopamine transmission in the mesolimbic pathway contributes to hallucinations, delusions, and cognitive distortions (14). The dopamine hypothesis of psychosis suggests that heightened dopamine activity leads to aberrant salience processing, wherein neutral stimuli are misinterpreted as overly significant, fostering delusional thought patterns (15). Neuroimaging studies have revealed increased presynaptic dopamine synthesis in individuals with psychosis, further supporting its role in the pathophysiology of this disorder (14). Additionally, dopamine hypersensitivity in psychotic disorders has been linked to abnormal sensory perception, reinforcing perceptual distortions (16, 17).

The potential connection between migraine and psychosis may be explained by shared dopaminergic dysfunction. Both conditions involve dopamine receptor hypersensitivity, sensory disturbances, and altered cortical excitability (11, 16). Migraine with aura, in particular, has been associated with transient psychotic symptoms, including hallucinations, paranoia, and perceptual distortions, suggesting overlapping neurobiological mechanisms (3, 4). Dopamine–serotonin interactions may also underlie the co-occurrence of migraine and psychiatric disorders, as serotonergic disruptions can modulate dopaminergic activity, impacting both pain perception and psychotic symptoms (4, 11).

This review examines the role of dopamine in both migraine and psychosis, assessing whether dopaminergic dysfunction in migraineurs heightens the risk of psychotic symptoms. While both conditions share dopamine-related abnormalities, their precise relationship remains uncertain. By synthesizing neurobiological, clinical, and pharmacological evidence, this review critically evaluates existing research, highlights unresolved questions, and outlines future directions to clarify the migraine–psychosis link. Understanding this connection may provide insight into shared neurobiological mechanisms and inform the development of targeted therapeutic approaches for both disorders.

## Migraine pathophysiology and the dopaminergic system

The trigeminovascular system plays a key role in migraine pathophysiology, mediating pain transmission through trigeminal afferents that project to the trigeminocervical complex (TCC) and further to higher brain centers (2, 6). Dopamine modulates this system by acting on trigeminal nociceptive pathways, with studies indicating that the A11 dopaminergic nucleus projects to the TCC and inhibits trigeminovascular activation (2, 6, 8). The dopaminergic system, particularly the A11 dopaminergic nucleus, plays a critical role in modulating trigeminal pain pathways. Studies have demonstrated that increased dopaminergic receptor binding in regions such as the striatum and insula is correlated with sensory hypersensitivity and increased pain perception during migraine attacks (18). This receptor hypersensitivity is not only implicated in migraine but also a hallmark of psychotic disorders, reinforcing the shared neurobiological underpinnings between both conditions. Disruptions in dopamine signaling may lead to increased pain sensitivity and contribute to migraine attacks (9, 11, 19). Migraineurs exhibit hypersensitivity to dopamine agonists, which manifests as enhanced responses to apomorphine, leading to nausea, yawning, and vomiting (9, 10). This hypersensitivity suggests an altered dopaminergic response, potentially linked to increased D2 receptor sensitivity (7, 8). Dopaminergic dysregulation may also contribute to the sensory disturbances observed in migraine patients, such as photophobia and allodynia (11).

Dopamine influences sensory processing by modulating pain perception in key brain regions, including the periaqueductal gray, nucleus accumbens, and spinal cord (10, 11, 19). The interaction between dopamine and serotonin also plays a role in migraine pathophysiology, with imbalances in these neurotransmitters affecting the pain threshold and cortical excitability (7, 8). Studies indicate that dopamine levels fluctuate throughout migraine phases, with decreased dopaminergic activity during attacks potentially leading to hypersensitivity and increased pain perception (7, 8, 11). Evidence also suggests that migraineurs have altered dopamine metabolism, which could contribute to recurrent attacks and associated symptoms (9). These findings highlight the integral role of dopamine in migraine pathophysiology and its potential link to other neuropsychiatric conditions.

## Dopaminergic system and psychotic disorders

Dopaminergic dysfunction plays a critical role in the pathophysiology of psychotic disorders (20). The dopamine hypothesis of psychosis postulates that excessive dopamine transmission, particularly in the mesolimbic pathway, contributes to delusions and hallucinations (14, 21). Neuroimaging studies have revealed increased presynaptic dopamine synthesis in individuals with psychosis, reinforcing its role in psychotic symptomatology (14). Individuals with psychotic disorders often exhibit dopamine receptor hypersensitivity, leading to heightened responsiveness to stimuli (16). This results in aberrant salience attribution, reinforcing delusional thinking (15). Schizophrenia and other psychotic disorders are associated with increased D2 receptor density, reinforcing a link between dopaminergic overactivity and cognitive distortions (16).

Studies have established a clear link between dopamine dysfunction and psychosis. The aberrant salience hypothesis suggests that excessive dopamine activity enhances the significance of unrelated stimuli, leading to misinterpretations and delusions (15). Functional imaging studies have demonstrated dopamine dysregulation in early-stage psychosis, further reinforcing its role in psychotic pathology (14).

## Shared neurobiological mechanisms between migraine and psychotic disorders

**Figure 1:**
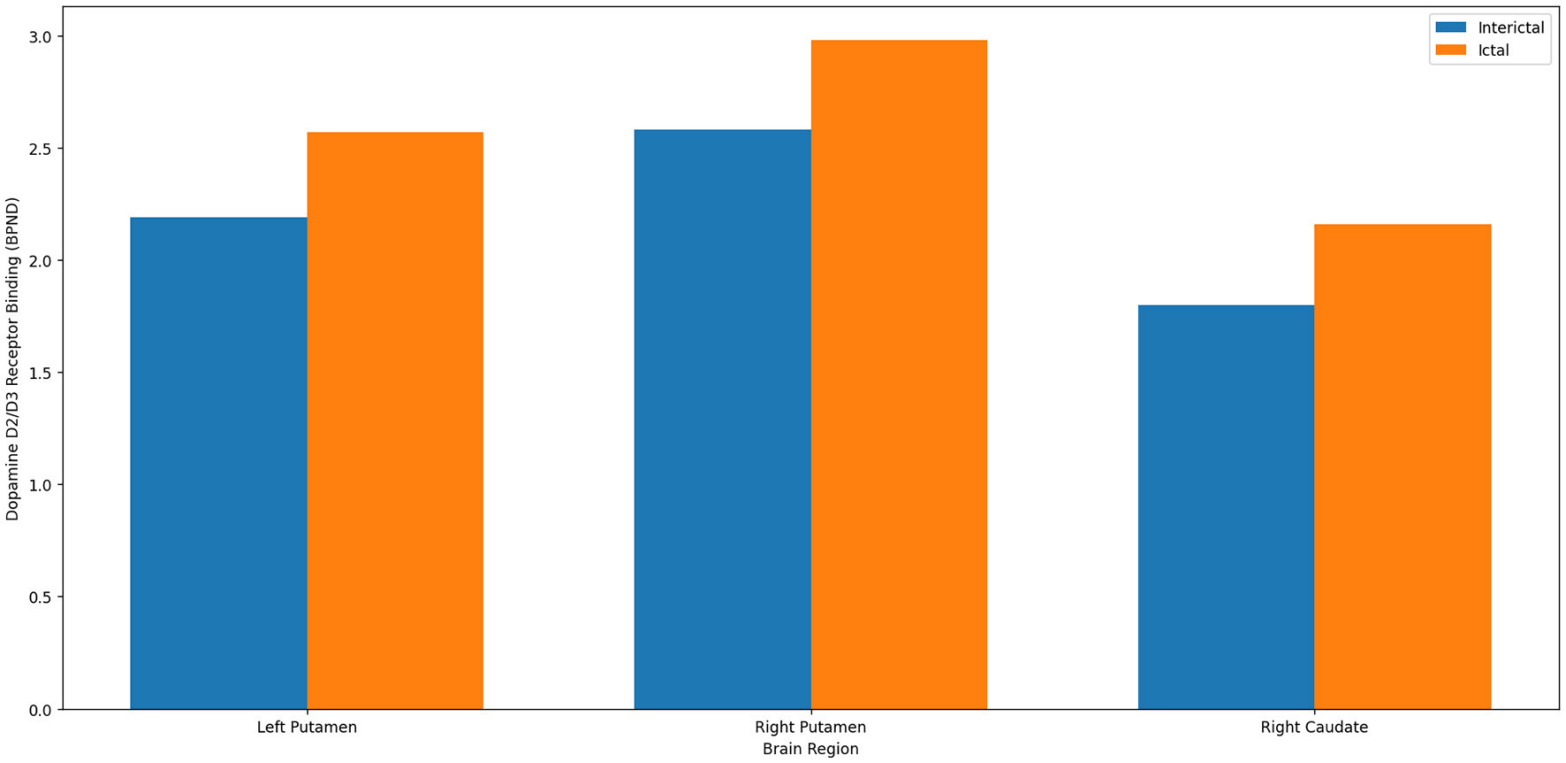
Comparison of dopamine D2/D3 receptor binding potential (BPND) during the interictal and ictal migraine phases. Data adapted from DaSilva et al. (18).

**Figure 2:**
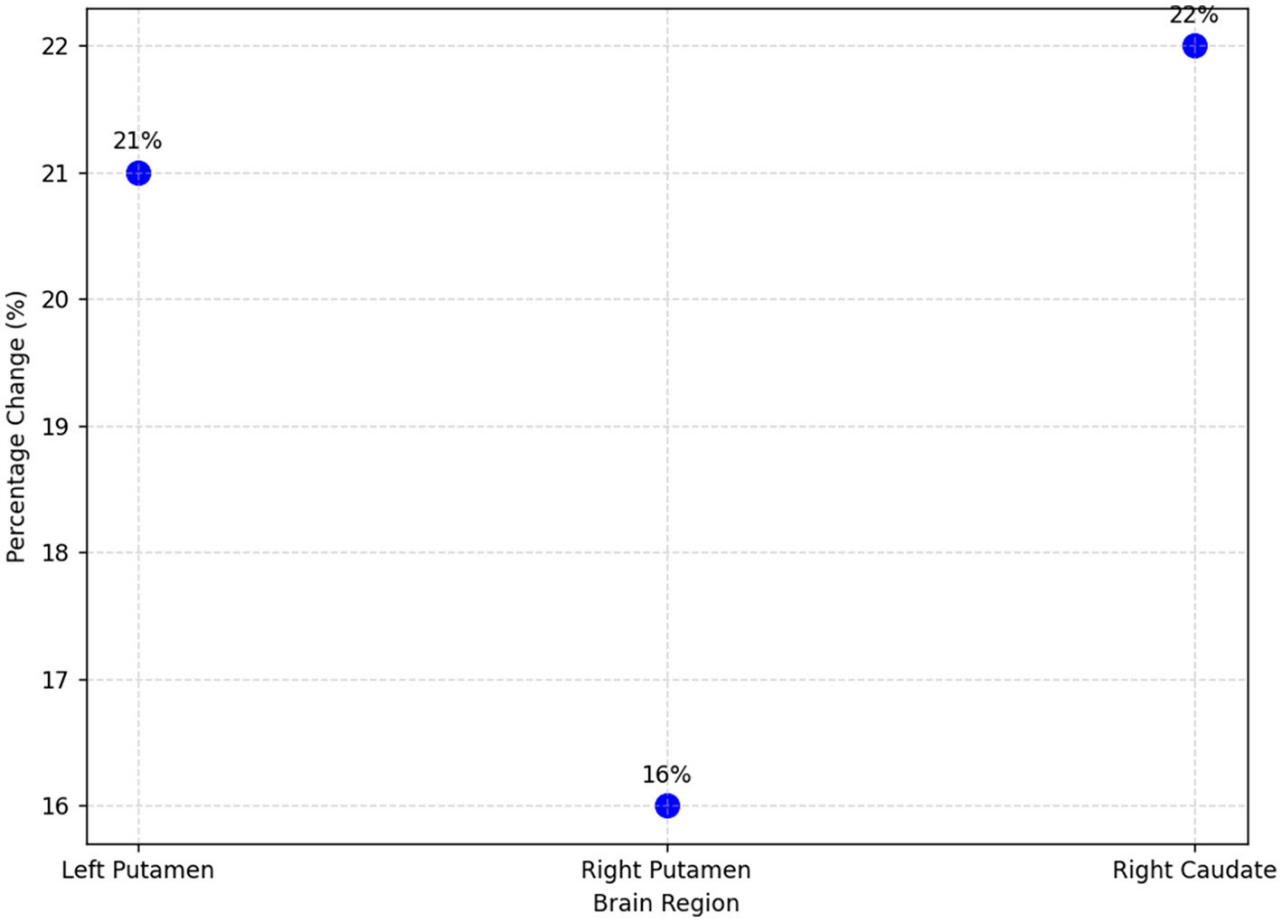
Percentage changes in dopamine D2/D3 receptor binding potential during migraine attacks across brain regions. Data adapted from DaSilva et al. (18).

Dopamine dysfunction is implicated in both migraine and psychotic disorders. Recent neuroimaging studies have also revealed altered dopamine receptor activity in key regions, such as the prefrontal cortex and basal ganglia, during migraine attacks, which overlaps with the brain regions associated with psychosis (22). While migraine with aura is linked to transient psychotic symptoms such as paranoia and hallucinations, the specific mechanisms behind these phenomena remain unclear. However, they could be a result of transient alterations in the dopaminergic system that overlap with the aberrant salience processing observed in psychotic disorders (14). Migraineurs exhibit dopaminergic hypersensitivity, characterized by increased responsiveness to dopamine agonists, resulting in symptoms such as nausea, yawning, and hypotension induced by apomorphine (9, 10). Recent neuroimaging studies highlighted significant dopamine fluctuations during migraine episodes, particularly by positron emission tomography (PET) imaging, which revealed increased dopamine D2/D3 receptor binding, reflecting decreased dopamine release during attacks (18, 23) (see Figure 1 for a clear comparison of dopamine receptor binding between the interictal and ictal migraine phases). Increased dopamine D2/D3 receptor binding potential (BPND), as demonstrated by PET scans during migraine attacks, indicates that fewer endogenous dopamine molecules are available to occupy receptor sites, directly suggesting a reduction in dopamine release during these episodes (18). These changes are consistent with receptor hypersensitivity and heightened sensory responsiveness. These dopamine variations likely contribute to the sensory disturbances commonly observed in migraine, such as photophobia and allodynia (7, 8, 11) (refer to Figure 2, which illustrates the percentage changes in dopamine receptor binding during migraine attacks across specific brain regions). Furthermore, dopamine plays a central role not only in pain modulation but also in the regulation of sensory perception and salience attribution, functions that are commonly disrupted during migraine attacks. Studies have suggested that alterations in dopaminergic tone may transiently affect reality processing, potentially leading to misperceptions, derealization, and illusion-like phenomena, particularly during the ictal or aura phases of migraine (4, 8, 11). While these disturbances do not represent full-blown psychosis, they share qualitative similarities with psychotic symptoms and may explain why some migraineurs, especially those with aura, report transient perceptual anomalies. This dopaminergic contribution to sensory misattribution may serve as a mechanistic link between migraine and psychotic-like experiences without implying the development of psychotic disorders.

While dopamine dysfunction is implicated in migraine pathophysiology irrespective of aura status, psychotic-like symptoms have been described predominantly in patients with migraine with aura. Patients with migraine without aura (MWoA) exhibit dopaminergic hypersensitivity, often reflected by premonitory symptoms such as nausea, yawning, and somnolence during migraine attacks (8). However, frank psychotic manifestations such as hallucinations and delusions are rarely reported in this subgroup. This finding suggests that while dopaminergic dysfunction contributes to sensory and pain symptoms in both migraine types, additional mechanisms, possibly related to cortical spreading depression and altered cortico-subcortical processing, may play crucial roles in the occurrence of transient psychotic-like symptoms primarily observed in migraine patients with aura (18, 22).

**Figure 3:**
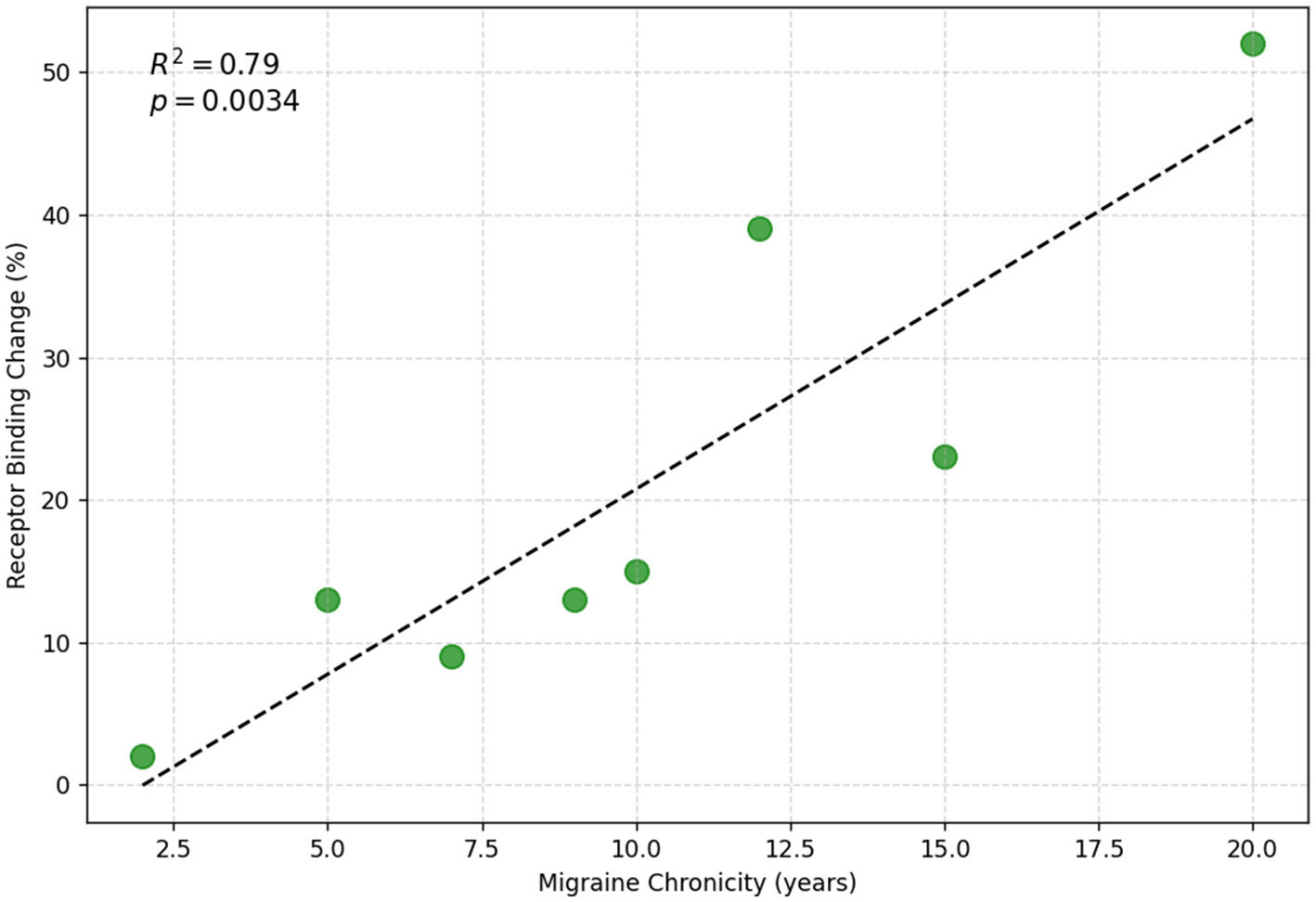
Correlation between migraine chronicity and changes in dopamine D2/D3 receptor binding potential (BPND). Data adapted from DaSilva et al. (18).

Given the critical role of dopamine in sensory perception, pain modulation, and cognitive processing, disruptions in dopaminergic function may bridge migraine symptoms and psychotic manifestations (3). It is crucial to acknowledge the heterogeneity of migraine subtypes and psychiatric disorders. Different migraine subtypes, particularly migraine with aura and migraine without aura, exhibit varying degrees of dopaminergic dysfunction. Additionally, psychiatric comorbidities in migraine patients vary, with some showing a higher prevalence of transient psychotic symptoms than others do, possibly due to underlying genetic or environmental factors (3). Dopamine receptor hypersensitivity and dynamic neurotransmitter fluctuations in migraineurs can lead to sensory amplification, mirroring the aberrant salience processing observed in psychotic disorders, which is driven by excessive dopamine activity within the mesolimbic pathway and results in perceptual distortions and delusional interpretations (24). Increased dopaminergic activity in individuals with migraine, especially chronic migraineurs or those experiencing frequent aura episodes, may predispose them to transient psychotic-like symptoms (25, 26). Figure 3 shows the correlation between migraine chronicity and dopamine receptor binding changes.

**Figure 4:**
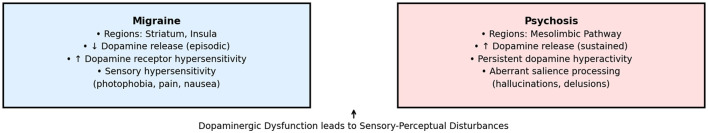
Conceptual schematic illustrating the differential patterns of dopaminergic dysfunction in migraine and psychosis. This figure was developed by the authors based on a synthesis of PET imaging findings, pharmacological data, and clinical observations from multiple studies, including DaSilva et al., Marino et al., and Howes and Kapur (14, 18, 23). It summarizes episodic dopamine hypoactivity with receptor hypersensitivity observed in migraine (especially during ictal phases), contrasted with sustained dopaminergic hyperactivity typically seen in psychotic disorders, particularly within the mesolimbic pathway.

Moreover, interactions between the dopamine and serotonin systems appear crucial in understanding the comorbidity between migraine and psychiatric disorders (11). Alterations within these neurotransmitter pathways significantly influence mood regulation, sensory integration, and cognitive interpretation, aspects central to both migraine and psychotic disorders (4). Neuroimaging findings further reinforce the common involvement of dopamine, revealing overlapping alterations in dopamine synthesis, receptor density, and neurotransmitter interactions under both conditions (14, 22). Although both migraine and psychotic disorders share dopaminergic dysfunction, it is important to note that the pattern of dysfunction differs between them. In migraine, dopaminergic dysfunction appears episodic, with fluctuations in dopamine release during migraine attacks, whereas psychotic disorders, particularly schizophrenia, are characterized by sustained dopaminergic hyperactivity, especially in the mesolimbic pathway (14). This difference in the pattern of dopaminergic dysfunction helps to explain why migraine may contribute to transient psychotic-like symptoms but not to full-blown psychotic disorders. In migraine, there is evidence of episodic reductions in dopamine release during attacks, along with receptor hypersensitivity, particularly in regions such as the striatum and insula, which contributes to sensory hypersensitivity and pain amplification (18, 23). In contrast, psychosis is characterized by sustained dopaminergic hyperactivity, particularly within the mesolimbic pathway, leading to perceptual distortions and aberrant salience processing (14). Recognizing this distinction is essential to understanding how migraine may contribute to transient psychotic symptoms without directly mimicking the pathophysiology of primary psychotic disorders. This distinction is summarized schematically in Figure 4, a conceptual illustration developed by the authors based on multiple sources, to highlight the contrast in dopaminergic dysfunction patterns between migraine and psychosis. Considering these overlapping neurobiological pathways, it is pertinent to further investigate whether migraine inherently increases the risk of developing psychotic symptoms. Recognizing the differential dopaminergic dysfunction patterns between migraine and psychotic disorders may help explain the emergence of transient psychotic-like symptoms in migraine patients without implying the development of full psychotic syndromes. Future studies employing advanced neuroimaging and longitudinal assessments are warranted to further elucidate this interaction.

## Can migraine increase the risk of psychosis?

Emerging research suggests that migraine may predispose individuals to psychotic symptoms, particularly those experiencing migraine with aura (3). Migraine with aura, marked by transient sensory disturbances and visual hallucinations, may share neurobiological mechanisms with psychotic perceptual distortions (4). The overlap in dopaminergic hypersensitivity and sensory misattributions suggests that migraineurs, particularly those with frequent auras, may be at increased risk of transient psychotic episodes (27). While direct causal evidence remains limited, clinical observations suggest a shared neurobiological predisposition (11). Furthermore, a previous study (4) confirmed that migraineurs experience higher rates of psychiatric comorbidities, including mood disorders, anxiety, and transient psychotic symptoms, than non-migraine individuals do. Functional imaging findings suggest that migraine-related alterations in dopamine metabolism may contribute to distorted reality perception, increasing vulnerability to psychotic states (14, 28).

## Clinical studies supporting the migraine-psychosis relationship

Several clinical studies provide evidence for a potential link between migraine and psychosis. Fornaro (4) highlighted that individual with bipolar disorder and comorbid migraine experience higher rates of psychotic symptoms, suggesting a shared pathophysiology. A 2021 cohort study of 500 migraineurs revealed that those with chronic migraine had twice the risk of developing transient psychotic symptoms compared with controls (3). Radat (3) examined migraine-associated psychiatric disturbances and demonstrated that migraineurs exhibit increased susceptibility to cognitive distortions and sensory misattributions, which may contribute to transient psychotic symptoms. A study conducted by Semiz et al. (29) examining 1,601 university students revealed that 23.1% of migraine patients had a current psychiatric diagnosis, with a significant proportion experiencing mood disturbances, anxiety disorders, and other psychiatric comorbidities. The study further revealed that migraine patients had a 43.2% lifetime prevalence of psychiatric disorders, suggesting a long-term neuropsychiatric burden associated with migraine. These findings indicate that chronic migraine may contribute to persistent alterations in neurobiological processes, potentially increasing susceptibility to psychiatric symptoms owing to prolonged dopaminergic dysregulation and shared pathophysiological mechanisms. Although this study was conducted on a relatively small-scale university cohort, its findings provide empirical support for the link between migraine and psychiatric disorders. The reported 43.2% lifetime prevalence of psychiatric conditions among migraine patients underscores a significant neuropsychiatric burden (29). Additionally, neuroimaging studies have revealed dopamine system abnormalities in both migraine and psychotic disorders, with heightened presynaptic dopamine activity in migraineurs paralleling findings in individuals with psychosis (14). These findings collectively suggest that migraine, particularly when accompanied by dopaminergic dysregulation, may contribute to neurocognitive alterations that increase the risk of psychotic episodes.

## Overlap in treatment strategies for migraine and psychosis

Dopamine antagonists play crucial roles in both migraine and psychotic disorder management, further reinforcing the hypothesis of shared dopaminergic dysfunction between these conditions (30). In migraine treatment, dopamine antagonists such as prochlorperazine, metoclopramide, and domperidone have been shown to alleviate headache symptoms, nausea, and sensory hypersensitivity, particularly in acute migraine attacks (31). These agents are thought to work by modulating dopamine pathways involved in pain perception, cortical excitability, and trigeminovascular activation, processes that are also implicated in psychotic disorders (32). Similarly, dopamine D2 receptor antagonists form the backbone of antipsychotic therapy in schizophrenia and other psychotic disorders, reducing delusions and hallucinations by normalizing excessive dopaminergic signaling in the mesolimbic system (33). Given that both migraine and psychotic disorders exhibit dopamine hypersensitivity and receptor dysfunction, the therapeutic efficacy of dopamine antagonists in both conditions supports the idea that a common neurobiological pathway contributes to their pathophysiology.

Furthermore, atypical antipsychotics that act on both dopamine and serotonin receptors (e.g., olanzapine and quetiapine) have been explored for migraine prophylaxis, with some reports suggesting their potential in reducing attack frequency in refractory cases (31). This finding aligns with the dopamine–serotonin interaction hypothesis, which proposes that dysregulation of these neurotransmitters contributes to both migraine and psychosis. The evidence supporting dopamine antagonists in both conditions strengthens the argument for a dopaminergic link between migraine and psychotic disorders. This shared pharmacological approach not only provides clinical justification for further research into dual treatment strategies but also highlights the importance of investigating the role of dopamine in sensory processing, pain modulation, and cognitive alterations across both disorders.

## Conclusion

The association between migraine and transient psychotic-like symptoms is increasingly supported by evidence of shared dopaminergic dysfunction. Migraine, particularly in those with aura, is characterized by dopamine hypersensitivity, neurotransmitter fluctuations, and sensory–perceptual disturbances—features that resemble key elements of psychotic disorders. The interaction between the dopaminergic and serotonergic systems further reinforces this link by highlighting common neurochemical mechanisms contributing to sensory amplification and altered perception. Clinical studies consistently report a higher prevalence of psychiatric comorbidities, including mood disturbances, anxiety, and transient psychotic-like symptoms, among migraineurs. Moreover, chronic migraine may predispose individuals to neuropsychiatric changes through persistent dopaminergic dysregulation and altered cortical excitability. Neuroimaging findings, such as elevated presynaptic dopamine activity during migraine and psychosis, provide neurobiological support for this association. The therapeutic effectiveness of dopamine antagonists under both conditions further supports the concept of a shared dopaminergic mechanism. However, while migraine may increase vulnerability to transient psychotic symptoms, this does not necessarily imply progression to a primary psychotic disorder. Early recognition and management of neuropsychiatric symptoms in migraineurs could prevent escalation into more severe complications. Understanding this relationship offers new avenues for integrated treatment strategies that address both neurological and psychiatric aspects, ultimately improving patient outcomes.

## Discussion

The growing body of research linking migraine and psychosis underscores the importance of dopaminergic dysfunction in both conditions. While both disorders involve dopamine hypersensitivity and neurotransmitter fluctuations, whether dopamine abnormalities are a primary cause of migraine or a secondary consequence of broader neurochemical disruptions remains debated. Some researchers argue that dopaminergic dysfunction directly contributes to migraine pathophysiology, whereas others suggest that it arises through serotonergic and CGRP-related mechanisms (7, 8, 11). This controversy highlights the need for longitudinal neuroimaging studies to clarify whether dopamine dysregulation precedes or follows other neurochemical disturbances. Not all migraineurs exhibit dopaminergic hypersensitivity, and not all psychotic patients report migraine, suggesting that genetic, hormonal, or environmental factors may modulate susceptibility. Furthermore, many studies rely on self-reported psychiatric symptoms rather than structured clinical assessments, increasing the risk of misclassification (29). Future studies should prioritize large-scale, population-based cohorts with standardized diagnostic criteria. The therapeutic overlap between migraine and psychotic disorders presents both opportunities and challenges. D2 receptor antagonists, such as metoclopramide and prochlorperazine, effectively alleviate migraine symptoms but pose risks, including extrapyramidal symptoms (EPSs) and tardive dyskinesia (31, 32). Similarly, atypical antipsychotics (e.g., olanzapine and quetiapine) have been explored for migraine prophylaxis but carry risks of weight gain and metabolic syndrome, limiting their feasibility for routine migraine management. Given these concerns, dopaminergic treatments should be reserved for refractory cases, with serotonergic agents and CGRP inhibitors remaining first-line options. While some studies suggest that dopamine dysfunction may play a central role in migraine pathophysiology, it remains unclear whether it is a primary driver or a secondary consequence of broader neurochemical imbalances. Future research is needed to dissect these interactions more precisely. Notably, the dopaminergic modulation of salience, sensory perception, and cognitive appraisal may explain why some migraineurs, particularly those with aura, report transient psychotic-like experiences such as perceptual distortions, derealization, or illusions, even in the absence of a full psychotic disorder. This hypothesis is supported by imaging and clinical studies showing altered dopaminergic activity during migraine phases (4, 8, 11, 14), but future longitudinal studies are needed to confirm this association. This conceptual framework is visually summarized in Figure 4, which was developed by the authors through synthesis of multimodal evidence across PET imaging, pharmacological, and clinical studies.

## Future directions and clinical implications

To refine our understanding of the migraine–psychosis connection, future research should prioritize well-controlled, longitudinal studies to investigate whether the frequency and duration of migraine episodes are associated with the onset of psychotic-like symptoms over time, especially in those with frequent aura. Understanding the dopaminergic dysfunction that links migraine and psychosis could have significant clinical implications. Early identification of psychiatric symptoms in migraine patients, particularly those with frequent auras, could lead to preventive interventions, reducing the risk of psychotic episodes. Targeted therapies that modulate dopamine receptor activity, such as dopamine antagonists, may not only alleviate migraine symptoms but also prevent the development of psychotic symptoms in predisposed individuals (32). Advanced neuroimaging techniques could provide deeper insights into the shared dopaminergic dysfunction in both disorders, helping elucidate whether this shared dysregulation contributes to psychotic-like symptoms in migraineurs. Future studies should also aim to identify biomarkers that predict psychiatric risk in migraine patients, enabling early interventions. Additionally, personalized medicine approaches—tailoring migraine treatment to an individual's dopamine profile—could help mitigate psychiatric complications. Longitudinal studies on dopamine dysregulation in migraine may also reveal whether dopaminergic dysfunction precedes or follows other neurochemical disturbances. Considering the episodic yet impactful dopaminergic dysfunction observed in migraine patients, especially in those with aura, future studies should investigate whether these transient neurochemical alterations are sufficient to induce psychotic-like phenomena without leading to persistent psychotic syndromes. Longitudinal neuroimaging studies specifically correlating dopamine dynamics with transient psychotic symptoms in migraineurs are warranted to further clarify this potential overlap. Finally, controlled trials investigating whether migraine treatments influence psychiatric symptomatology in migraineurs are essential for optimizing treatment strategies. In addition, genetic studies could help identify biomarkers that predict susceptibility to both migraine and psychosis, allowing for early intervention in high-risk individuals. Clinically, recognizing the psychiatric risks associated with migraine may inform treatment strategies. Screening migraine patients for psychiatric symptoms, particularly those with frequent auras, could identify individuals at risk for psychotic-like symptoms and allow early intervention to prevent further neuropsychiatric complications. Neurologists and psychiatrists should collaborate in developing personalized treatment plans that minimize the risks of dopaminergic therapies while addressing both migraine and psychiatric comorbidities. In conclusion, while evidence suggests a strong neurobiological link between migraine and psychotic disorders, further research is essential to clarify causality and optimize treatment approaches. The potential therapeutic overlap between these conditions highlights the importance of an integrated, multidisciplinary approach to patient care, ensuring that both neurological and psychiatric aspects are adequately addressed. As the field advances, future reviews may benefit from a larger set of visual data from multiple imaging studies, enabling the development of more integrative illustrations grounded in multimodal evidence.
